# Assessment of Electromagnetic Field Exposure on European Roads: A Comprehensive In Situ Measurement Campaign

**DOI:** 10.3390/s23136050

**Published:** 2023-06-30

**Authors:** Gabriela Lachezarova Atanasova, Blagovest Nikolaev Atanasov, Nikolay Todorov Atanasov

**Affiliations:** 1 Department of Communication and Computer Engineering, South-West University “Neofit Rilski”, 2700 Blagoevgrad, Bulgaria; natanasov@swu.bg; 2 Faculty of Telecommunications, Technical University of Sofia, 1000 Sofia, Bulgaria; b_atanasov@outlook.com

**Keywords:** electromagnetic field, measurement, in situ measurement campaign, assessment, car

## Abstract

The rapid evolution of wireless communication technologies (such as fifth-generation (5G) cellular networks) in the last years has allowed connecting different objects (from wearable electronics to vehicles) and people through communication networks, and at the same time, has led to widespread deployment of base stations. Along with this growth, questions about the potential adverse effects on human health due to electromagnetic fields (EMFs) from base station antennas have also been raised. In this paper, we focus on the assessment of EMFs in automobiles during short (between cities) and long (between countries) trips on several European roads. Comprehensive measurement campaigns were carried out in several European countries: Austria, Bulgaria, Croatia, Hungary, Italy, Slovenia, and the Republic of Serbia. The results show that the median total electric field is 0.23–0.24 V/m in Bulgaria, Croatia, Hungary, Italy, and the Republic of Serbia. In Austria and Slovenia, the median is 0.28–0.31 V/m. Austria demonstrated the highest value for the total electric field, at 17.4 V/m.

## 1. Introduction

Mobility and connectivity are vital for our modern daily life [1]. In 2021 in Europe, Asia-Pacific, and the Americas, more than 90 percent of the population had access to a mobile broadband network [2] and digital services. According to the preliminary data reported in [2], the coverage of fifth-generation (5G) cellular networks reached 52 percent in Europe and 19 globally in 2021. The upcoming deployment of the next-generation cellular networks (such as 5G and 6G) and Internet-of-Things (IoT) will allow, in addition to mobile end-user devices (such as smartphones and tablets), vehicles, household appliances, wearable devices, and industrial machinery to be connected to the Internet and exchange data, which will contribute significantly to the improvement of healthcare services and road safety [3,4,5], and introduce new services for better resource use and lower greenhouse gas emissions [6].

The growth development of 5G cellular networks is also associated with the widespread deployment of base stations [7]. Along with this growth, questions also arise about the potential adverse effects on human health due to electromagnetic fields (EMFs) from base station antennas [8,9,10]. To debate this question, the European Parliament (Scientific and Technology Options Assessment Panel) organized a workshop on “Health and Environmental Impacts of 5G” [11]. Several papers have also focused on EMF assessment in various microenvironments, such as indoor [9,12,13] and outdoor (rural and urban) environments [8,14,15,16]. The data from the five largest monitoring networks in five different European countries show that exposure is below the International Commission on Non-Ionizing Radiation Protection (ICNIRP) reference level [17], and the median electric field value in the different countries varies between 0.67 V/m and 1.51 V/m [8]. A systematic review of the recent literature on the characterization of everyday human exposure to EMFs in European countries found that the mean electric field value in homes, schools, and offices varies between 0.04 and 0.76 V/m [18,19]. The highest peaks of electric field measured in shopping malls varies between 1.96 and 3.26 V/m [20]. Moreover, according to [18], the highest levels, up to 1.97 V/m, were measured in public transport stations. Several studies have investigated the electric field within urban public transport [21,22]. According to the presented results in [22], it can be concluded that the electric field value depends on the passengers’ distribution in the tram and the position of the EMF source.

Moreover, 5G mobile networks will play a key role in providing vehicular connectivity. A new concept known as “vehicle-to-everything (V2X)” has been developed, aiming to make transport safer, less polluting, and more convenient for passengers [23]. V2X communication encompasses vehicle-to-vehicle (V2V), vehicle-to-infrastructure (V2I), vehicle-to-pedestrian (V2P), and vehicle-to-network (V2N) communications [5,24,25]. Furthermore, 5G and 6G will also improve the existing in-car generic wireless communication services (e.g., in-cabin multimedia transmission, reliable and high-speed internet connection, etc.). Some new vehicles are already equipped with In-Car Wi-Fi Hotspots that allow connections of up to seven devices (laptops, tablets, etc.) [26,27] by embedded 5G connectivity.

New V2X and in-car generic wireless communications will lead to novel exposure scenarios for drivers and car passengers [5]. The presence of multiple sources of electromagnetic fields (such as Wi-Fi hot spots, mobile phones, Bluetooth hands-free, etc.) inside and outside the vehicle cabin has been suggested to lead to increasing EMFs inside the vehicle [28] due to the semi-resonant nature of the vehicle body [29,30].

Several studies have investigated through numerical simulations the electric field distribution within a vehicle cabin [29] and the relationship between the electric field exposure and the local (10 g on average) and whole-body average specific absorption rate (SAR) [5,28,30] in a human model of the car driver position at different frequencies (1.0 GHz, 1.5 GHz, 2.0 GHz, 2.4 GHz, 3.0 GHz, and 5.9 GHz). The antennas used in numerical simulations are resonant dipole [30], half-wave dipole [28,29], quarter-wave monopole [29], and ‘single edge’ voltage source [5]. Refs. [27,29] have reported that the maximum values of SAR are obtained in the planes corresponding to the chest and the head of the car driver. Moreover, in [5], the exposure to EMFs of the car driver was assessed in a realistic V2V communication scenario. A limited number of works have considered EMF exposure in urban areas using outdoor drive test measurements [31,32].

Moreover, there are no studies of EMFs in a car cabin during travel. According to the data presented in [33], in almost all European countries, the car is the dominant mode of transport for commuting to work and school, and people spend around 55 min every day in their vehicles [34]. Data presented in [35] show that in 2018, travel by car accounted for 82.9% of passenger kilometers across the European Union. In addition, more than 40% of people in Europe travel to a different country for recreation or a vacation [36]. Consequently, more studies are needed to estimate EMF exposure in a car during travel.

In our study, we measured the electric field in a vehicle cabin during short (between cities) or long (between countries) trips on several European roads (E60, E61, E65, E70, E75, and E80), with the expectation that these data may be helpful for adequate and accurate exposure assessment of both car driver and passengers. Our objective was to evaluate the electric field in the car cabin during driving on urban routes and motorways to investigate the relationship between different roads and electric field strength. We performed measurements in seven European countries: Austria, Bulgaria, Croatia, Hungary, Italy, Slovenia, and the Republic of Serbia in the period April 2022–April 2023. The total distance over which the measurements were carried out was more than 1900 km. Finally, a comparison of the measured values of the electric field in each of the seven countries is presented. We also present information for the field strength of *Ex*, *Ey*, and *Ez* components as an indicator of the orientation of the electric field, which is valuable for the design of both new absorbers of EMF for the car interior and new flexible rectennas for EMF energy harvesting in vehicles.

## 2. Materials and Methods

**Figure 1:**
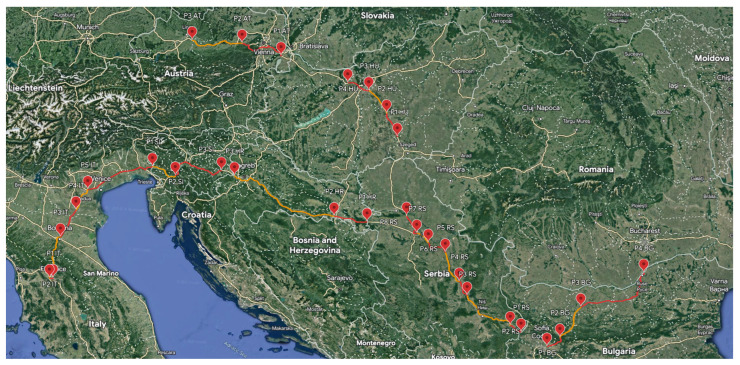
European roads selected to conduct measurements of the EMFs.

The roads selected for the in situ measurement campaign involve motorways and main roads, almost all of which are part of the international E-road network, as shown in Figure 1. All measurements were carried out inside a small car (Suzuki Celerio, a five-seat car with a 1.0 L three-cylinder turbocharged petrol engine and a 5-speed manual transmission) during short (up to 100 km) or long (exceeding 100 km) trips.

### 2.1. Measurement Equipment

A laser-powered electric field measurement system (LSProbe 1.2 Field Probe Variant E [37]) was used in all measurements. The system has high accuracy (±0.6 dB, ±1.0 dB, and ±1.4 dB in the frequency ranges of 9–30 MHz, 30–1 GHz, and 1–8.2 GHz, respectively), high speed (up to 2 MS/s), and high dynamic range (100 dB). Moreover, the LSProbe 1.2 Field Probe has a complete set of calibration data (measurement uncertainties are 12% in the frequency range of 0.03 to 1 GHz, and 18% from 1 to 8 GHz). The laser-powered electric field measurement system consists of an isotropic tri-axial antenna (or “probe’’), a measurement unit (CI-250), and a laptop. The electric field (E-field) probe records the E-field values using logarithmic power detectors and analog-to-digital converters and transmits the digital sample values to the CI-250, which was connected to the probe with a fiber optic cable. The laptop was connected to the CI-250 via a USB cable and was used as a host computer for the LSProbe TCP Server and for storing the measured data. The LSProbe TCP Server implements data post-processing and communication and also applies calibration data. The LSProbe Graphical User Interface software was used to manage the probe settings, data acquisition, and storage. The measured data (field strength of the *x*-, *y*- and *z*-axis and the field strength magnitude) were recorded in a log file every 500 ms. During the measurements, the GPS coordinates of each point were recorded by a GPS logger. Table 1 presents the settings of the LSProbe 1.2 Field Probe Variant E used during all measurements.

### 2.2. Measurement Procedure

A number of points (P1, P2, etc.) were defined on each road. On the map (see Figure 1), each point is labeled as P, followed by the point number and country abbreviation (for example, point 1 in Bulgaria is labeled as P1 BG). The measurement system was set up to measure the electric field strength (in the frequency range of 0.03 GHz to 8.2 GHz for the *x*-, *y*-, and *z*-axis) between each of the two points, i.e., in one road sector, as shown in Table 2. During the measurements, the car kept a steady speed of 110 km/h (on motorways and main roads) and 50 km/h (in towns and villages). The results of each measurement (for example, between points P1 AT–P2 AT or P2 AT–P3 AT) together with the GPS coordinates of each point were stored in separate data sets. The total distance over which the measurements were carried out was more than 1900 km.

All measurements were taken, for one year, between April 2022 and April 2023, using the same car. During all measurements, the E-field probe was mounted on the car’s back seat on Styrofoam at the height of the passenger’s neck (at a distance of 50 cm from the car’s roof and 40 cm from the nearest window. Only a driver and a passenger (operator of the measurement equipment in the front right seat) were in the car cabin. Moreover, during the measurements, the mobile phones of the driver and passenger were shut down so as to not contribute to the data. In addition, the car’s infotainment system (including built-in Bluetooth) was turned off.

Table 2 presents the driving distance (in km) on each road sector (between two points) in each country.

Data from all measurements were processed and analyzed.

### 2.3. Data Acquisition, Processing, and Analysis

As described in the previous subsections, the electric field strengths of the *x*-, *y*- and *z*-axis and the field strength magnitude (total electric field) were recorded by the LSProbe 1.2 Field Probe Variant E, with an interval of 500 ms.

The processing and aggregation of the large data set were performed using OriginPro2018 software. A non-parametric Kolmogorov–Smirnov test was used to test the normality of the distribution in each data set. We found that the data do not have a normal distribution. The mean, median, and standard deviation for each data set were calculated. We used the interquartile range (IQR) to measure the data variability because IQR is less sensitive to extreme values. To represent both the summary statistics and the distribution of the data, boxplots (box-and-whisker plots) of the data were constructed. This data representation also allows the identification of outliers in the data set.

## 3. Results and Discussion

In this section, the results of electric field strength (V/m), which were measured in the car during travel on different roads in seven European counties, are presented and discussed. The mean, median, minimum, and maximum values of measurements in each road sector are listed in Table 3.

### 3.1. Electromagnetic Field on Different European Roads

#### 3.1.1. Austria

**Figure 2:**
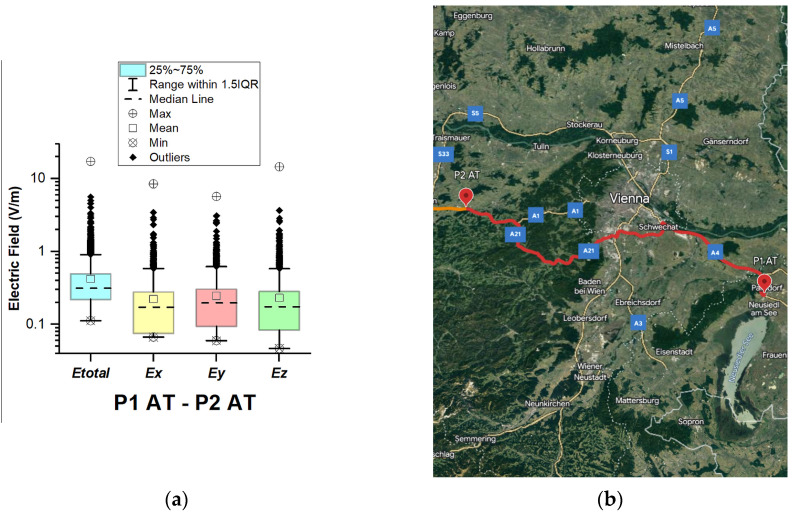
Austria: (**a**) Boxplot showing total electric field (*E_tota_*_l_) and electric field on each axis (*E_x_, E_y_,* and *E_z_*). The central dotted line in the box shows the median, and the bottom and top edges are the 25th and 75th percentiles, respectively; (**b**) Map showing the selected road on which measurements were taken.

**Figure 3:**
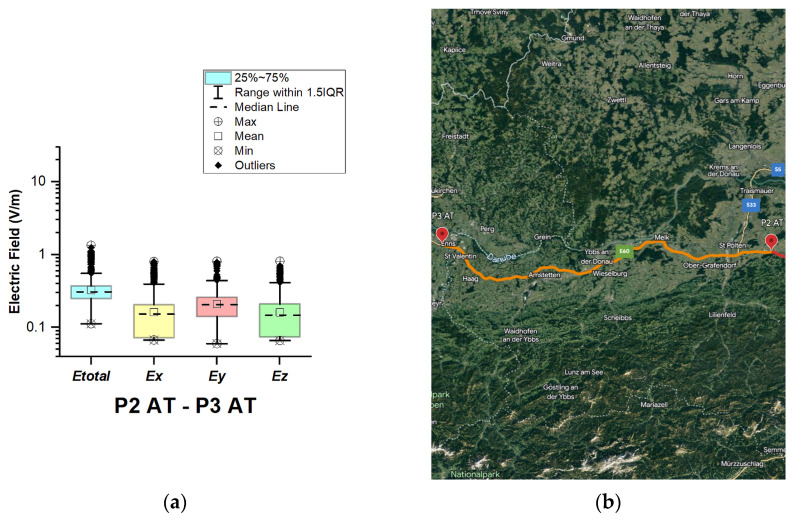
Austria: (**a**) Boxplot showing total electric field (*E_total_*) and electric field on each axis (*Ex*, *Ey*, and *Ez*). The central dotted line in the box shows the median, and the bottom and top edges are the 25th and 75th percentiles, respectively; (**b**) Map showing the selected road on which measurements were taken.

In Austria, electric field measurements were carried out in the car cabin during driving on both motorways and expressways (between points P1 AT–P2 AT and P2 AT–P3 AT), as shown in Figure 2 and Figure 3. The road section of P1 AT–P2 AT covers part of the motorways (A4 and A21) and expressway S1 (see Figure 2b). P2 AT–P3 AT covers part of the A1 motorway. The selected driving roads are part of the second longest road in the International E-road network—E 60.

From Figure 2 and Figure 3 and Table 3, it can be seen that the median of *E_total_* is 0.31 V/m for the two road sections. In addition, from the box plots, we can see that in the first road section (P1 AT–P2 AT) the mean is 0.42 V/m; this indicates that the data are skewed. Moreover, there are outliers in the distribution for this road section (P1 AT–P2 AT), and a maximal value of 17.4 V/m is obtained. The components of the E-field for the maximal value are 8.5 V/m (*Ex*), 5.7 V/m (*Ey*), and 14.6 V/m (*Ez*), which can be used as an indicator for the orientation of the E-field of the incident electromagnetic wave. We assume that obtained results reflect that part of the Vienna Ring Road falls within this road section. Another factor that contributes to the higher electric field value is the deployment of the base station antennas. In the first road section (P1 AT–P2 AT), *Ex*, *Ey*, and *Ez* components have almost the same mean (*Ex* = 0.22 V/m, *Ey =* 0.24 V/m, and *Ez* = 0.23 V/m) and median (*Ex* = 0.17 V/m, *Ey =* 0.19 V/m, and *Ez* = 0.17 V/m), meaning that each of the components contributes equally to *E_total_* due to the multipath environment of the electromagnetic wave propagation in the car’s cabin. From Figure 3, we can see that the electric field on the probe *y*-axis has a slightly higher mean and median in the second road section (P2 AT–P3 AT). The distribution of the electromagnetic field in the car depends on the intensity and polarization of the incident and reflected electromagnetic waves. In the road section where the measurements were made, electromagnetic waves probably penetrated through the car side windows. We suppose that the higher measured values at the *y*-axis (placed parallel to the side windows) of the LSProbe variant E are caused by the arrival of the direct signal from the side windows.

#### 3.1.2. Bulgaria

**Figure 4:**
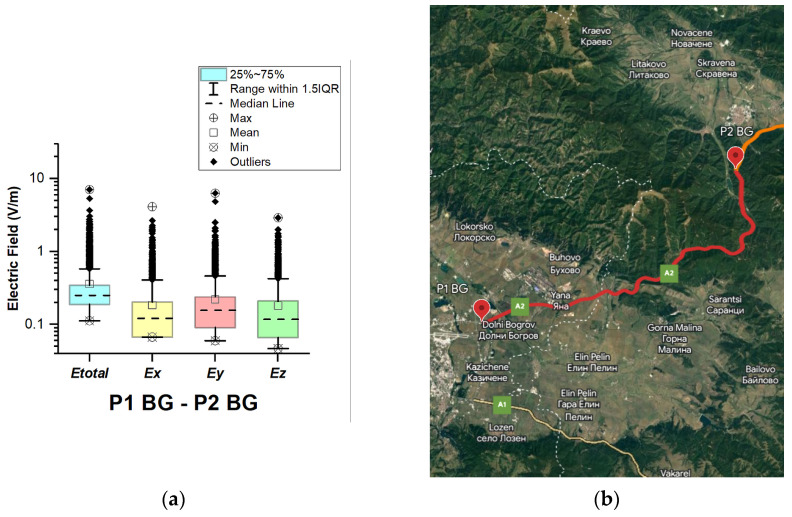
Bulgaria: (**a**) Boxplot showing total electric field (*E_tota_*_l_) and electric field on each axis (*E_x_, E_y_,* and *E_z_*). The central dotted line in the box shows the median, and the bottom and top edges are the 25th and 75th percentiles, respectively; (**b**) Map showing the selected road on which measurements were taken.

**Figure 5:**
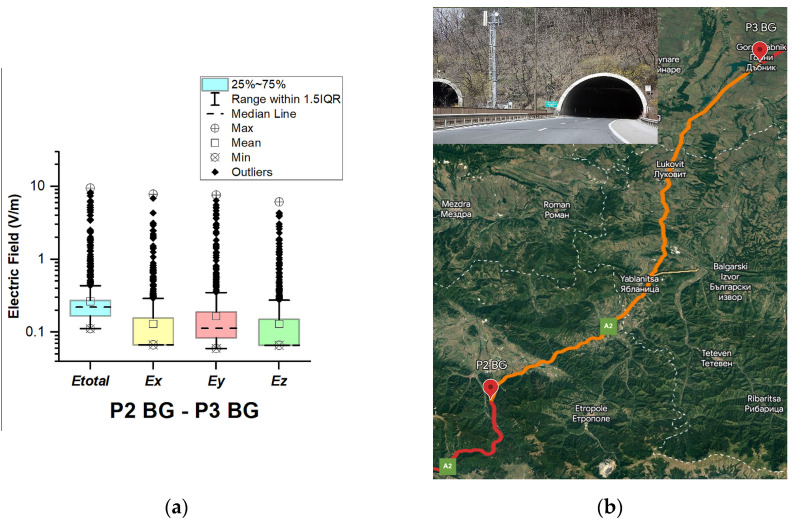
Bulgaria: (**a**) Boxplot showing total electric field (*E_tota_*_l_) and electric field on each axis (*E_x_*, *E_y_*, and *E_z_*). The central dotted line in the box shows the median, and the bottom and top edges are the 25th and 75th percentiles, respectively; (**b**) Map showing the selected road on which measurements were taken.

**Figure 6:**
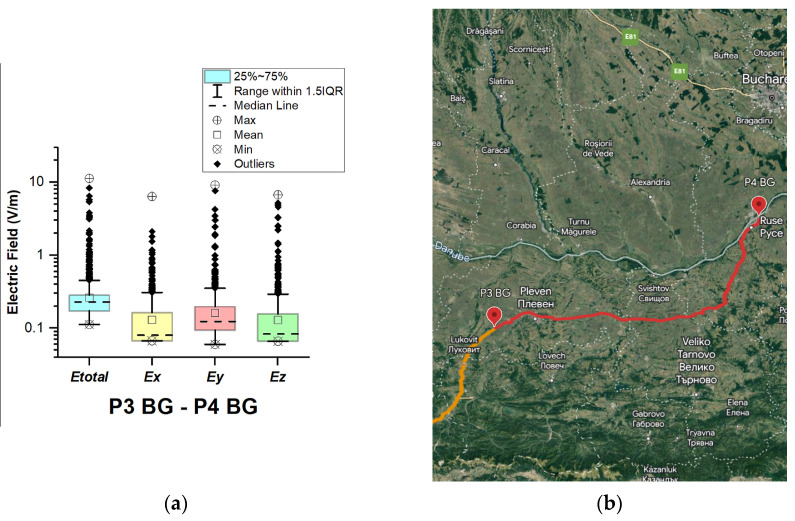
Bulgaria: (**a**) Boxplot showing total electric field (*E_total_*) and electric field on each axis (*E_x_*, *E_y_*, and *E_z_*). The central dotted line in the box shows the median, and the bottom and top edges are the 25th and 75th percentiles, respectively; (**b**) Map showing the selected road on which measurements were taken.

In Bulgaria, EMF measurements were carried out in the car cabin during driving on a motorway and a main road (between points P1 BG–P2 BG, P2 BG–P3 BG, and P3 BG–P4 BG), as shown in Figure 4, Figure 5 and Figure 6. The road sections between points P1 BG–P2 BG and P2 BG– P3 G cover part of the motorway A2. The third road section (between points P3 BG–P4 BG) is a main road that is part of the E83 and E85.

Figure 4, Figure 5 and Figure 6 and Table 3 show that the data do not have a normal distribution. From the boxplots, we can see that the distributions of the E-field in the car’s cabin during traveling on the different road sectors in Bulgaria are skewed: *E_total_* median is 0.25 V/m (P1 BG–P2 BG) and 0.22 V/m (P2 BG–P3 BG and P3 BG–P4 BG) and mean is 0.36 V/m (P1 BG–P2 BG) and 0.26 V/m (P2 BG–P3 BG and P3 BG–P4 BG). During driving on the third road section (P3 BG–P4 BG), we obtained the highest maximal *E_total_* value of 11.3 V/m. We suppose that the measured single high value of the electric field is due to the passage of the vehicle near EMF sources, such as base station antennas of telecommunication operators. From Figure 6b, it can be seen that the third road section passes through one of the largest cities in Bulgaria—Ruse. The two selected road sections of the A2 motorway do not pass through densely populated areas. However, the maximum obtained values of the electric field in the car’s cabin in these two road sections were also high: 7.1 V/m (P1 BG–P2 BG) and 9.6 V/m (P2 BG–P3 BG). Comparing these values with the maximum *E_total_* value on the A1 motorway in Austria (1.4 V/m, P2 AT–P3 AT), we can conclude that the maximum measured values when driving on the A2 motorway (P1 BG–P2 BG and P2 BG–P3 BG) in Bulgaria are higher. This may be due to the fact that there are base station antennas near the A2 motorway tunnels in Bulgaria, as shown in Figure 5b.

The lowest *E_total_* value in all road sections is 0.11 V/m.

#### 3.1.3. Croatia

**Figure 7:**
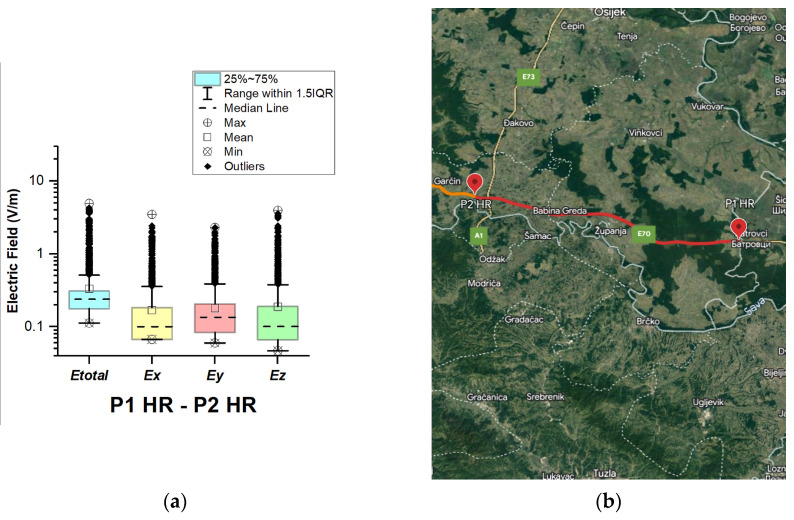
Croatia: (**a**) Boxplot showing total electric field (*E_total_*) and electric field on each axis (*E_x_*, *E_y_*, and *E_z_*). The central dotted line in the box shows the median, and the bottom and top edges are the 25th and 75th percentiles, respectively; (**b**) Map showing the selected road on which measurements were taken.

**Figure 8:**
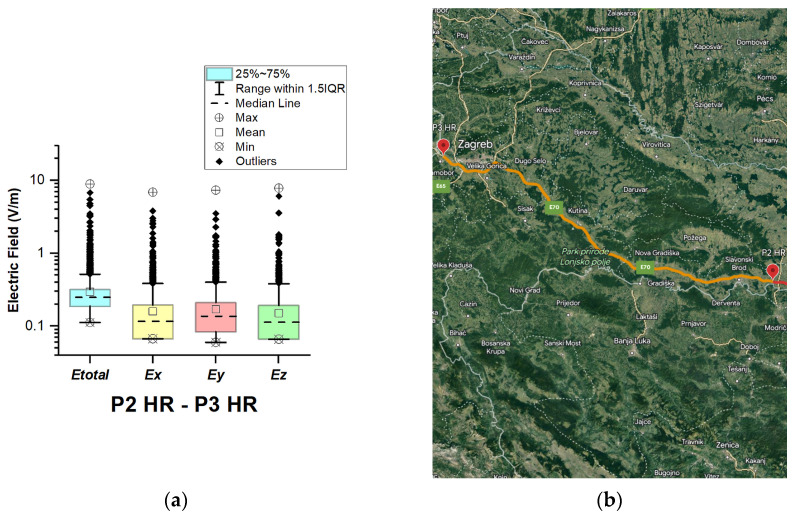
Croatia: (**a**) Boxplot showing total electric field (*E_total_*) and electric field on each axis (*E_x_*, *E_y_*, and *E_z_*). The central dotted line in the box shows the median, and the bottom and top edges are the 25th and 75th percentiles, respectively; (**b**) Map showing the selected road on which measurements were taken.

In Croatia, EMF measurements were carried out in the car cabin during driving on motorway A3 (in two road sections between points P1 HR–P2 HR and P2 HR–P3 HR), as shown in Figure 7 and Figure 8. The selected driving road is part of the International E-road network—E 70.

During driving on the second road section (P2 HR–P3 HR), the maximal value of the total electric field was 8.96 V/m. The obtained value is approximately twice as high as that in the section P1 HR–P2 HR. We assume that obtained results are because P3 HR is part of the Zagreb Ring Road. Another factor that contributes to the higher electric field value is the deployment of the base station antennas.

The median of *E_total_* is 0.24 V/m for road section P1 HR–P2 HR and 0.25 V/m for road section P2 HR–P3 HR. The lowest *E_total_* value in the two road sections was 0.11 V/m. We see that the *E_total_* upper quartile (Q3 = 0.31 V/m) is about the same for the two distributions. In addition, from Figure 7 and Figure 8, we can see that the electric field on the probe *y*-axis has a slightly higher median in the two road sections. We assume that the obtained results are caused by the distribution of the E-field inside the car, which varies due to the many different routes of propagation of electromagnetic waves from the transmitter/s to the LSProbe. The electromagnetic waves penetrate through windows and reflect off the car chassis. In addition, some electromagnetic waves penetrate the car’s seats. We suppose that measured single higher values of the electric field are due to the passage of the vehicle near EMF sources.

#### 3.1.4. Hungary

**Figure 9:**
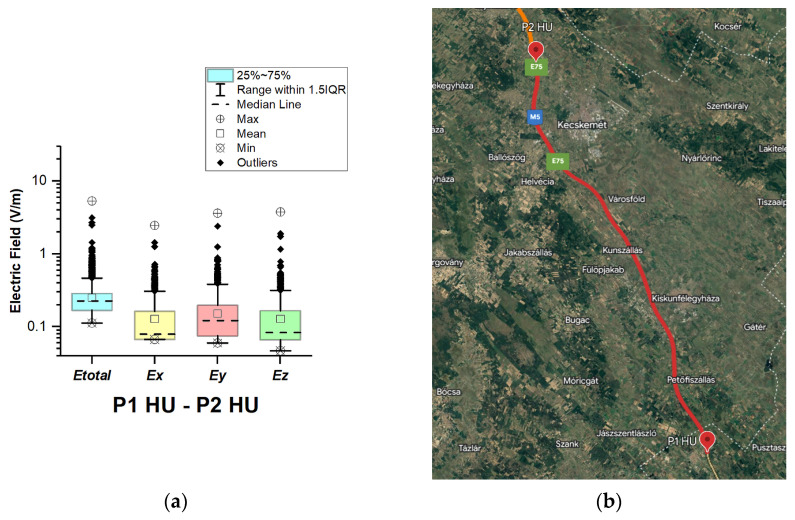
Hungary: (**a**) Boxplot showing total electric field (*E_total_*) and electric field on each axis (*E_x_*, *E_y_*, and *E_z_*). The central dotted line in the box shows the median, and the bottom and top edges are the 25th and 75th percentiles, respectively; (**b**) Map showing the selected road on which measurements were taken.

**Figure 10:**
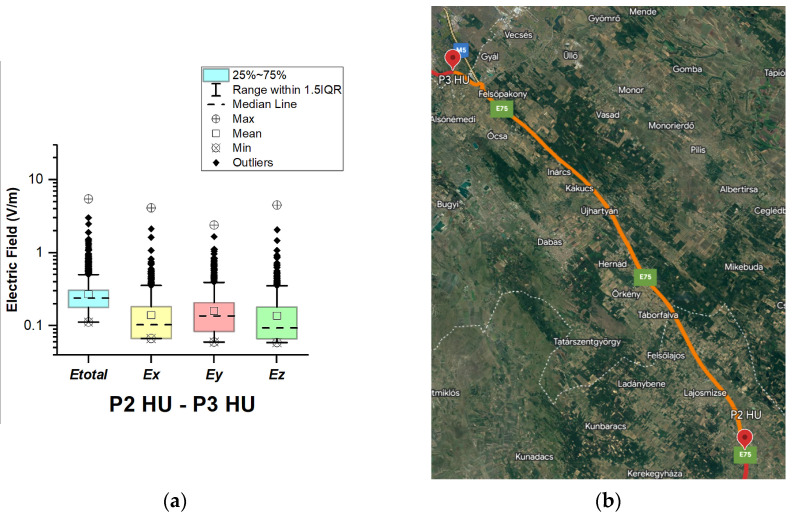
Hungary: (**a**) Boxplot showing total electric field (*E_total_*) and electric field on each axis (*E_x_*, *E_y_*, and *E_z_*). The central dotted line in the box shows the median, and the bottom and top edges are the 25th and 75th percentiles, respectively; (**b**) Map showing the selected road on which measurements were taken.

**Figure 11:**
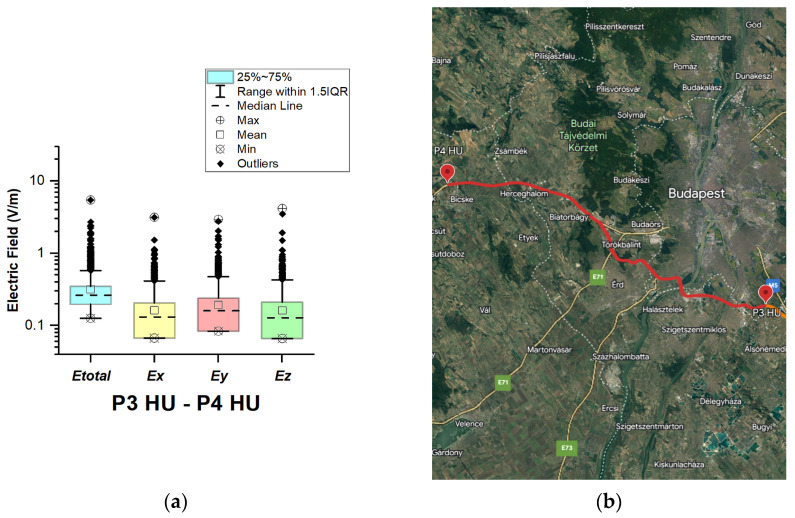
Hungary: (**a**) Boxplot showing total electric field (*E_total_*) and electric field on each axis (*E_x_*, *E_y_*, and *E_z_*). The central dotted line in the box shows the median, and the bottom and top edges are the 25th and 75th percentiles, respectively; (**b**) Map showing the selected road on which measurements were taken.

In Hungary, electric field measurements were carried out in the car cabin during driving on motorways M0, M1, and M5, as shown in Figure 9, Figure 10 and Figure 11. The selected driving roads are part of the International E-road network—E 75 (between points P1 HU–P2 HU and P2 HU–P3 HU) and E60 (P3 HU–P4 HU).

Figure 9, Figure 10 and Figure 11 and Table 3 show that the maximum *E_total_* values vary slightly from 5.33 V/m to 5.50 V/m. Although part of the road section P2 HU–P3 HU covers the Budapest Ring Road, no higher value was measured compared to the other two road sections. The median of *E_total_* is between 0.24 V/m and 0.26 V/m. The lowest *E_total_* value in all road sections is 0.11 V/m. We see that the *E_total_* upper quartile is 0.29 V/m (P1 HU–P2 HU), 0.31 V/m (P2 HU–P3 HU), and 0.35 V/m (P3 HU–P4 HU). Additionally, the electric field on the probe *y*-axis has a slightly higher mean and median. We suppose that obtained results are caused by the E-field distribution inside the car, which varies due to the many different routes of propagation of electromagnetic waves from the transmitter/s to the LSProbe. The signal that arrives at the probe axis is composed of direct and reflected components. The higher measured values at the *y*-axis of the LSProbe 1.2 variant E are likely caused by the direct component.

#### 3.1.5. Italy

**Figure 12:**
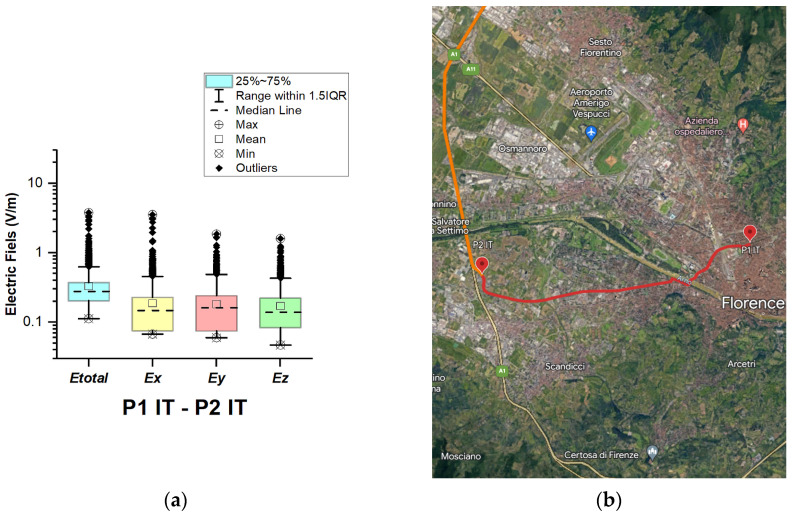
Italy: (**a**) Boxplot showing total electric field (*E_total_*) and electric field on each axis (*E_x_*, *E_y_*, and *E_z_*). The central dotted line in the box shows the median, and the bottom and top edges are the 25th and 75th percentiles, respectively; (**b**) Map showing the selected road on which measurements were taken.

**Figure 13:**
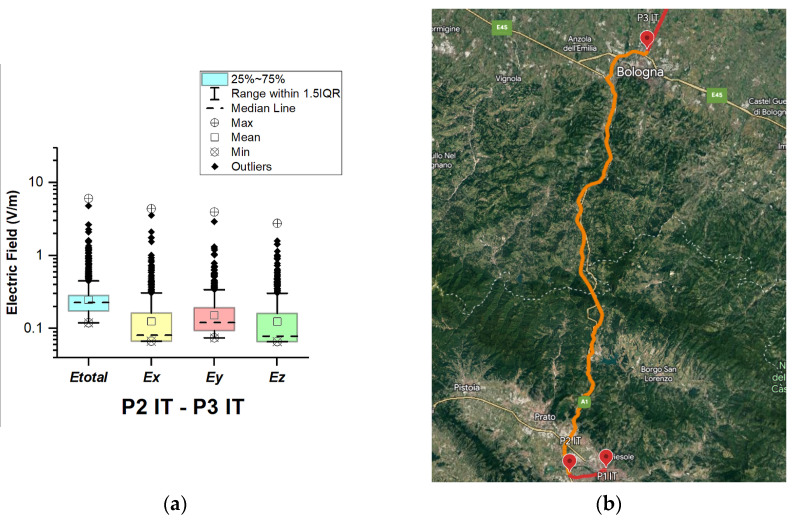
Italy: (**a**) Boxplot showing total electric field (*E_total_*) and electric field on each axis (*E_x_*, *E_y_*, and *E_z_*). The central dotted line in the box shows the median, and the bottom and top edges are the 25th and 75th percentiles, respectively; (**b**) Map showing the selected road on which measurements were taken.

**Figure 14:**
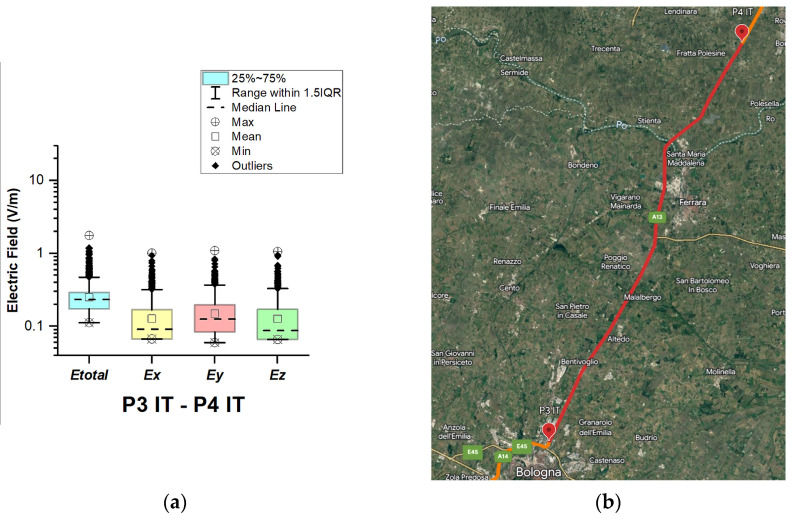
Italy: (**a**) Boxplot showing total electric field (*E_total_*) and electric field on each axis (*E_x_*, *E_y_*, and *E_z_*). The central dotted line in the box shows the median, and the bottom and top edges are 25th and 75th percentiles, respectively; (**b**) Map showing the selected road on which measurements were taken.

**Figure 15:**
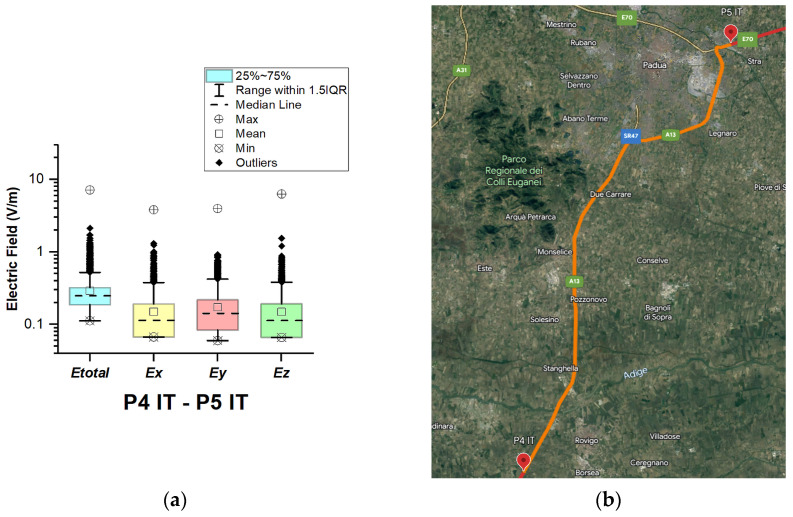
Italy: (**a**) Boxplot showing total electric field (*E_total_*) and electric field on each axis (*E_x_*, *E_y_*, and *E_z_*). The central dotted line in the box shows the median, and the bottom and top edges are the 25th and 75th percentiles, respectively; (**b**) Map showing the selected road on which measurements were taken.

**Figure 16:**
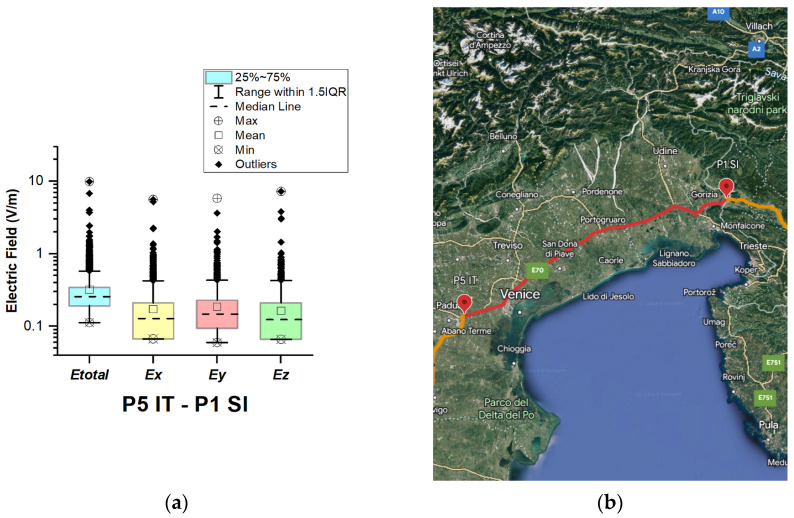
Italy: (**a**) Boxplot showing total electric field (*E_total_*) and electric field on each axis (*E_x_*, *E_y_*, and *E_z_*). The central dotted line in the box shows the median, and the bottom and top edges are the 25th and 75th percentiles, respectively; (**b**) Map showing the selected road on which measurements were taken.

In Italy, electric field measurements were carried out in the car cabin during driving on urban and main roads as well as motorways (A1, A13, A14, A34, and A57), as shown in Figure 12, Figure 13, Figure 14, Figure 15 and Figure 16.

The first road section, between points P1 IT–P2 IT, is only 8.3 km long, from Florence to the A1 motorway. The aim was to assess the exposure while driving on an urban road. Figure 12 and Table 3 show that the *E_total_* median and mean values in the car cabin are 0.28 V/m and 0.33 V/m, and the highest value is 3.8 V/m. The highest obtained value is close to that reported for shopping malls in [20], being lower than the values measured in road sections between points P2 IT–P3 IT, P4 IT–P5 IT, and P5 IT–P1 Sl.

From Figure 13, Figure 15 and Figure 16 and Table 3, it can be seen that when the road sections cover the ring roads of large cities such as Bologne, Padoue, etc., the measured maximum value of the electric field is high. The highest value for road sections P2 IT–P3 IT, P4 IT–P5 IT, and P5 IT–P1 Sl is 6.1 V/m, 7.2 V/m, and 9.4 V/m, respectively. From the results, we also see that the highest value for the road section P3 IT–P4 IT (see Figure 14) is 1.76 V/m. We suppose that obtained results are due to the fact that there are no EMF sources near the road, unlike the rest of the road sections, which cover ring roads of large cities and are near base station antennas of telecommunication operators.

The median of *E_total_* is between 0.23 V/m and 0.27 V/m. In almost all road sections, the lowest *E_total_* value is 0.11 V/m. The mean value in different road sections varies between 0.25 V/m and 0.32 V/m.

#### 3.1.6. Slovenia

**Figure 17:**
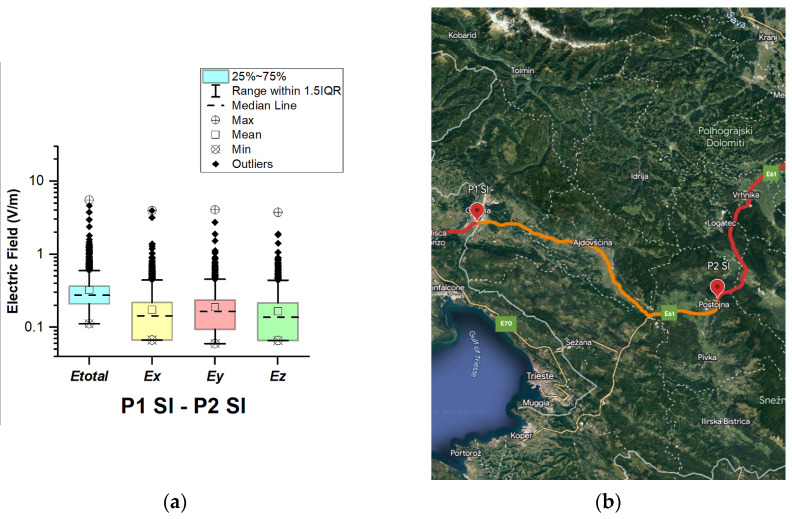
Slovenia: (**a**) Boxplot showing total electric field (*E_total_*) and electric field on each axis (*E_x_*, *E_y_*, and *E_z_*). The central dotted line in the box shows the median, and the bottom and top edges are the 25th and 75th percentiles, respectively; (**b**) Map showing the selected road on which measurements were taken.

**Figure 18:**
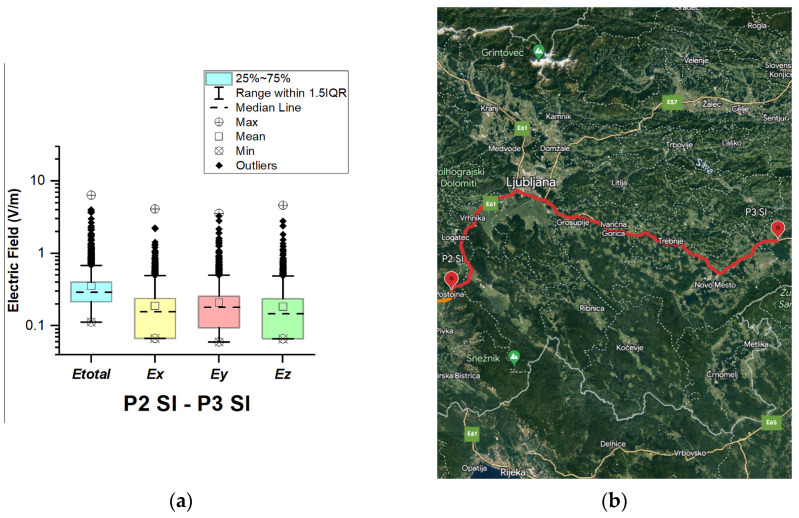
Slovenia: (**a**) Boxplot showing total electric field (*E_total_*) and electric field on each axis (*E_x_*, *E_y_*, and *E_z_*). The central dotted line in the box shows the median, and the bottom and top edges are the 25th and 75th percentiles, respectively; (**b**) Map showing the selected road on which measurements were taken.

In Slovenia, electric field measurements were carried out in the car cabin during driving on both motorways and expressways (between points P1 Sl–P2 Sl and P2 Sl–P3 Sl), as shown in Figure 17 and Figure 18.

The road section between points P1 Sl–P2 Sl covers part of the motorways (A34 and A1) and expressway H4. P2 Sl–P3 Sl covers part of the A1 and A2 motorways. The selected driving roads are part of the International E-road network–E 61 (between points P1 Sl–P2 Sl) and E70 (between points P2 Sl–P3 Sl).

The median of *E_total_* is 0.28 V/m for road section P1 Sl–P2 Sl and 0.29 V/m for road section P2 Sl–P3 Sl. The lowest *E_total_* value in the two road sections is 0.11 V/m. The highest *E_total_* values are 5.61 V/m and 6.46 V/m in the road sections P1 Sl–P2 Sl and P2 Sl–P3 Sl, respectively. Moreover, the results show that the *E_total_* upper quartile is 0.37 V/m (P1 Sl–P2 Sl) and 0.40 V/m (P2 Sl–P2 Sl). We assume that obtained results are due to the fact that part of the road section between points P2 Sl–P3 Sl covers the Ljubljana Ring Road; therefore, more cellular network antennas are available close to the roads, which is one of the factors in the higher *E_total_* values in the second road section.

#### 3.1.7. The Republic of Serbia

**Figure 19:**
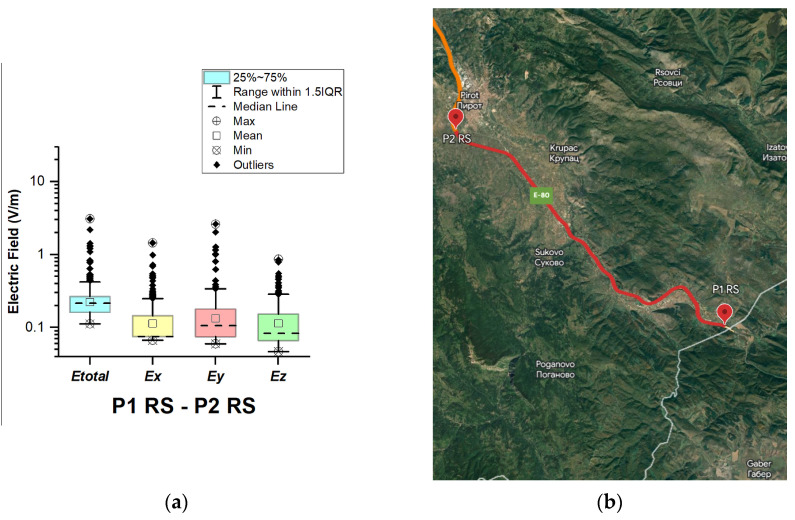
The Republic of Serbia: (**a**) Boxplot showing total electric field (*E_total_*) and electric field on each axis (*E_x_*, *E_y_*, and *E_z_*). The central dotted line in the box shows the median, and the bottom and top edges are the 25th and 75th percentiles, respectively; (**b**) Map showing the selected road on which measurements were taken.

**Figure 20:**
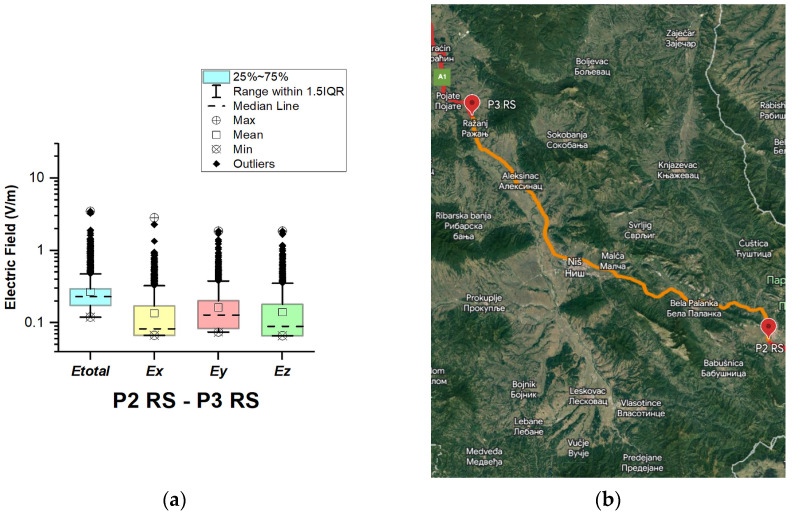
The Republic of Serbia: (**a**) Boxplot showing total electric field (*E_total_*) and electric field on each axis (*E_x_*, *E_y_*, and *E_z_*). The central dotted line in the box shows the median, and the bottom and top edges are the 25th and 75th percentiles, respectively; (**b**) Map showing the selected road on which measurements were taken.

**Figure 21:**
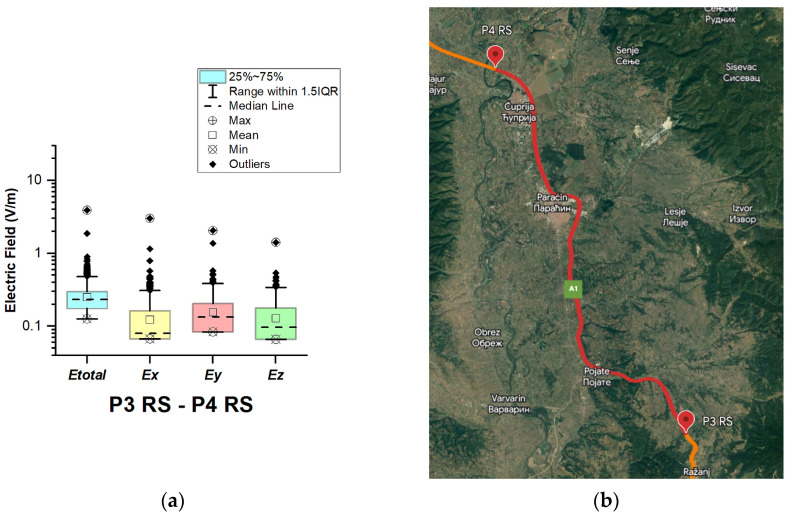
The Republic of Serbia: (**a**) Boxplot showing total electric field (*E_total_*) and electric field on each axis (*E_x_*, *E_y_*, and *E_z_*). The central dotted line in the box shows the median, and the bottom and top edges are 25th and 75th percentiles, respectively; (**b**) Map showing the selected road on which measurements were taken.

**Figure 22:**
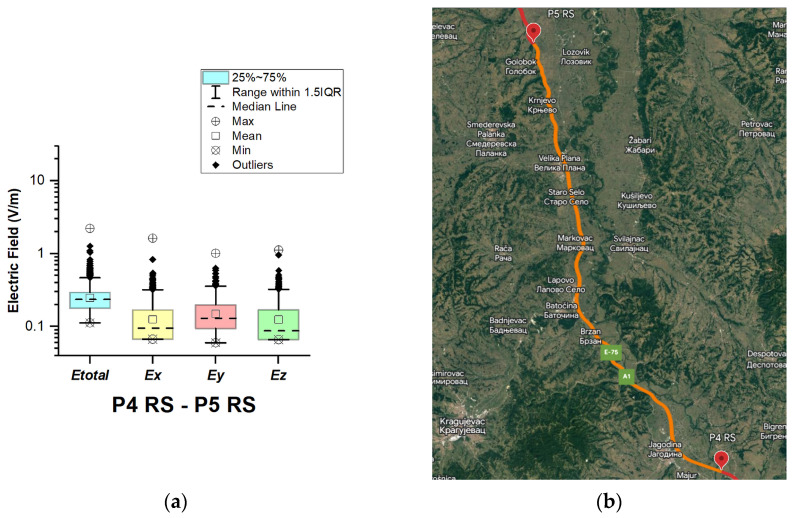
The Republic of Serbia: (**a**) Boxplot showing total electric field (*E_total_*) and electric field on each axis (*E_x_*, *E_y_*, and *E_z_*). The central dotted line in the box shows the median, and the bottom and top edges are the 25th and 75th percentiles, respectively; (**b**) Map showing the selected road on which measurements were taken.

**Figure 23:**
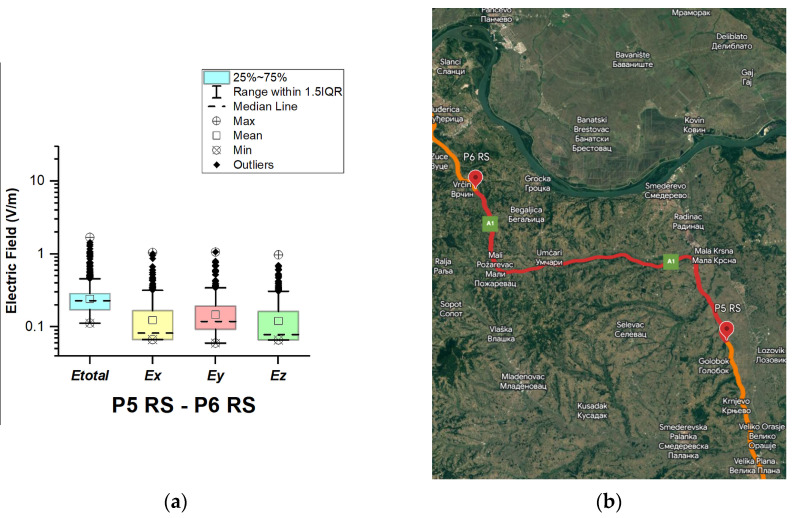
The Republic of Serbia: (**a**) Boxplot showing total electric field (*E_total_*) and electric field on each axis (*E_x_*, *E_y_*, and *E_z_*). The central dotted line in the box shows the median, and the bottom and top edges are the 25th and 75th percentiles, respectively; (**b**) Map showing the selected road on which measurements were taken.

**Figure 24:**
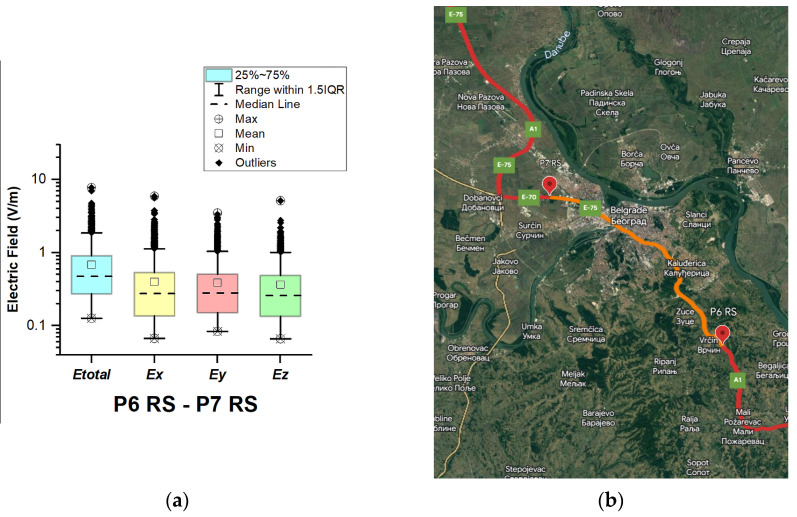
The Republic of Serbia: (**a**) Boxplot showing total electric field (*E_total_*) and electric field on each axis (*E_x_*, *E_y_*, and *E_z_*). The central dotted line in the box shows the median, and the bottom and top edges are the 25th and 75th percentiles, respectively; (**b**) Map showing the selected road on which measurements were taken.

**Figure 25:**
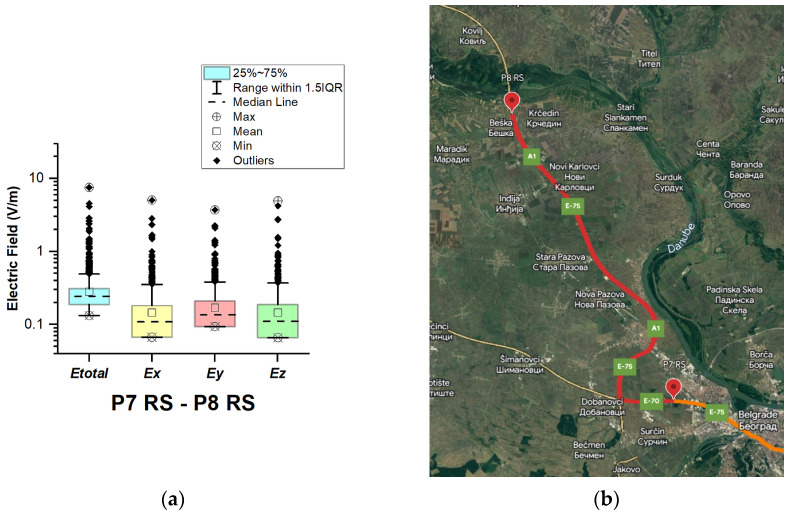
The Republic of Serbia: (**a**) Boxplot showing total electric field (*E_total_*) and electric field on each axis (*E_x_*, *E_y_*, and *E_z_*). The central dotted line in the box shows the median, and the bottom and top edges are the 25th and 75th percentiles, respectively; (**b**) Map showing the selected road on which measurements were taken.

In the Republic of Serbia, electric field measurements were carried out in the car cabin during driving on the motorways (A1 and A4), as shown in Figure 19, Figure 20, Figure 21, Figure 22, Figure 23, Figure 24 and Figure 25. The selected driving roads are part of the International E-road network: E 70, E75, and E80.

Figure 19 and Table 3 show that the *E_total_* mean value in the car’s cabin during driving on E80 is 0.22 V/m, the lower quartile is 0.16 V/m, the median is 0.21 V/m, and the upper quartile is 0.27 V/m. From the data in Figure 20, Figure 21 and Figure 22, we see that the lower quartile (Q1 = 0.18 V/m), median (Q2 = 0.23 V/m) and upper quartile (Q3 = 0.29 V/m) are about the same for the *E_total_* distributions in the car’s cabin during driving on the road sections on A1 motorways between points P2 RS–P3 RS, P3 RS–P4 RS, and P4 Rs–P5 RS. In the road section between points P6 RS–P7 RS, the highest value of *E_total_* was 7.76 V/m. The lower quartile (Q1 = 0.27 V/m), median (Q2 = 0.47 V/m), and upper quartile (Q3 = 0.91 V/m) in this road section were higher than the lower quartile, median, and upper quartile in other road sections in the Republic of Serbia. This road section passes through Belgrade, the capital of the Republic of Serbia. This is a very densely populated urban area in which there are a large number of sources of EMFs, such as the antennas of cellular network base stations near the roadway. In our opinion, this is the reason for higher measured EMF values for the three components of the E-field. The obtained results also show that in a very dense urban area, the electric field values are higher compared to rural ones. These observations are also confirmed by the results of other authors [38].

Analyzing the data from all measurements, we see that the mean and median of the E-field on the *y*-axis of LSProbe are slightly higher for the road sections presented in Figure 3, Figure 4, Figure 5, Figure 6, Figure 9, Figure 10, Figure 11, Figure 13, Figure 14, Figure 16, Figure 17, Figure 18, Figure 19, Figure 20, Figure 21, Figure 22, Figure 23 and Figure 24 compared to the E-field values on the other two axes. These results are due to the complex environment in which the electromagnetic waves propagate in the car cabin. The electric field measured by the LSProbe is a combination of both signals penetrated through windows and reflected off the inside of the vehicle body. When electromagnetic waves reflect off car interior surfaces, their polarization is changed. The fact that the E-field on the *y*-axis is slightly higher means that electromagnetic waves penetrating through the car side windows fall on the *y*-axis (placed parallel to the side windows). Electromagnetic waves that penetrate through the other car windows are reflected off interior surfaces and fall over on the antenna. These results may be applicable in the development of new materials for car interiors that have excellent electromagnetic wave absorption properties [39] and thus lead to a reduction of both passenger exposure and electromagnetic interference. The results will also apply to EMF energy harvesting, helping to more precisely determine the locations of the rectennas so that more EMF energy can be harvested in the vehicle during travel.

**Figure 26:**
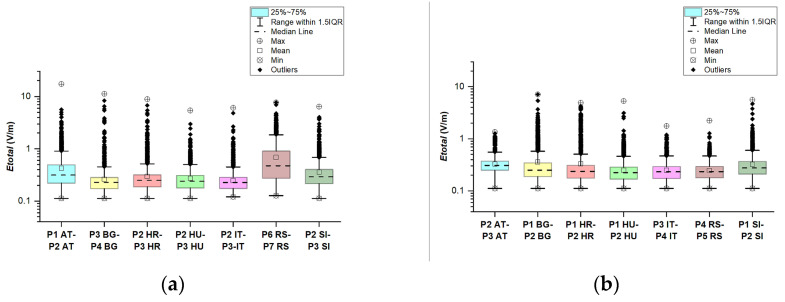
Comparison of the total electric field (*E_total_*) on (**a**) Motorways and main roads passing through large cities or covering ring roads of large cities; (**b**) Motorways and main roads that do not pass through populated areas.

From the data, we can see that higher maximal values of the electric field are observed in the car cabin when driving on roads that pass through large cities or ring roads of large cities. In Figure 26, a comparison between the different road sections is made. We see from Figure 26a that the distributions of *E_total_* in the car’s cabin during driving on the road sectors P1 AT–P2 AT and P6 RS–P7 RS have a higher median line than others. Moreover, the P1 AT–P2 AT and P6 RS–P7 RS medians are higher than the upper quartile for P3 BG–P4 BG, P2 HR–P3 HR, P2 HU–P3 HU, P2 IT–P3 IT, and P2 Sl–P3 Sl. Hence, we can conclude that the entire distributions of *E_total_* on P1 AT–P2 AT and P6 RS–P7 RS are shifted upwards relative to other road sectors. From the data in Figure 26b, we see that the lower quartile and median are about the same for the distributions of *E_total_* in the car’s cabin during driving on the road sectors from motorways or roads through small cities and villages: P1 HR–P2 HR, P1 HU–P2 HU, P3 IT–P4 IT, and P4 RS–P5 RS. In addition, the median for the distributions in P2 AT–P3 AT and P1 Sl–P2 Sl is higher than the median for P1 HR–P2 HR, P1 HU–P2 HU, P3 IT–P4 IT, and P4 RS–P5 RS. In [8], after analyzing data from five large monitoring networks for different microenvironments in five European countries, the authors conclude that electric field values increase with increasing population density. The obtained results in this work also show that in urban and suburban environments, the electric field values are higher compared to rural ones.

### 3.2. Comparison of Results between Different European Countries

**Figure 27:**
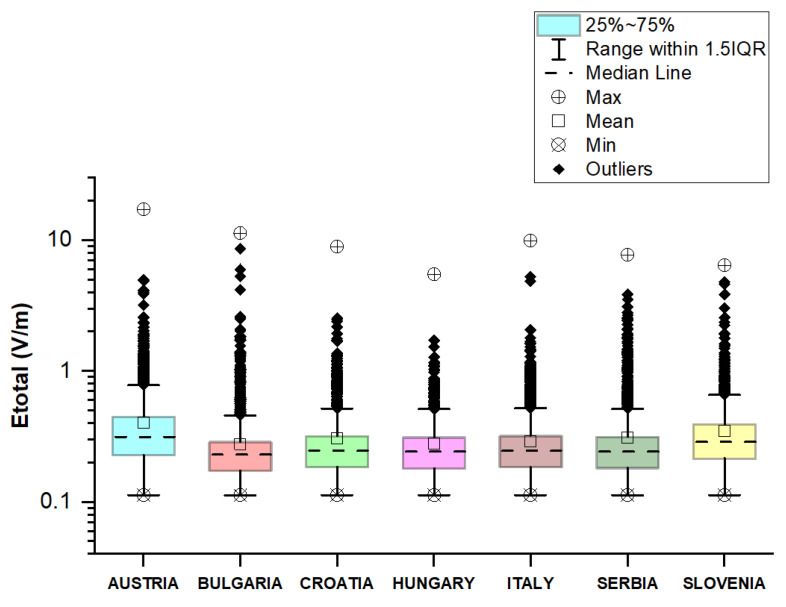
Comparison of the results between different European countries.

The measured values of the electric field for each country are summarized and presented in Figure 27. From the boxplots, we can see that the distributions of the *E_total_* in the car’s cabin during traveling on different roads in different countries are skewed. We see that the lower quartile (Q1 = 0.18 V/m) and median (Q2 = 0.24 V/m) are the same for *E_total_* distributions in Croatia, Hungary, Italy, and the Republic of Serbia. Moreover, *E_total_* distribution in Austria and the Republic of Serbia has more variability than in other countries. In addition, the highest value of 17.4 V/m was obtained in Austria on the Vienna Ring Road. We supposed that these results are due to the fact that two of the road sectors pass through Vienna and Belgrade, the capitals of Austria and the Republic of Serbia. These are the very densely populated urban areas in which there are a large number of sources of EMF, such as the antennas of cellular network base stations close to roadways. The total electric field in the car cabin during travel varied insignificantly across the different countries. The median of *E_total_* is 0.23 V/m in Bulgaria, 0.28 V/m in Slovenia, and 0.31 V/m in Austria.

Moreover, the data show that the exposure of car drivers and passengers during travel on European roads is below the ICNIRP reference level [17].

The obtained data are helpful for adequate and accurate exposure assessment of both car drivers and passengers during short or long travel. Moreover, the results of this work can be used to study human exposure to new 5G-V2X communication scenarios, such as vehicle-to-vehicle, vehicle-to-infrastructure, and vehicle-to-network wireless communications. The obtained results of this measurement campaign will have far-reaching applications, including electromagnetic compatibility (EMC), the development of new absorbers of EMF in cars, and the development of new flexible rectennas for EMF energy harvesting in vehicles. EMC testing for new vehicle components (sensors, ABS, airbags, etc.) is required by law in order to avoid an accident caused by electromagnetic influences on the onboard electronics. Another application where the results of E-field measurements in a car are desired for the development of new materials is for the car interior, where excellent electromagnetic wave absorption properties [39] can lead to reducing passenger exposure and electromagnetic interference. The results will also apply to EMF energy harvesting by helping to more precisely determine the locations of the rectennas so that more EMF energy can be collected in the vehicle during travel.

## 4. Conclusions

In conclusion, this paper provides for the first time results from the comprehensive in situ measurement of the EMFs in a car cabin during travel on part of several European roads (E60, E61, E65, E70, E75, and E80). The measurements were performed in seven European countries: Austria, Bulgaria, Croatia, Hungary, Italy, Slovenia, and the Republic of Serbia in the period April 2022–April 2023. The total distance over which the measurements were carried out was more than 1900 km.

We found that the higher maximal values of the electric field are observed in the car cabin when driving on roads that pass through large cities or ring roads of large cities. In Austria, the highest maximal value of 17.4 V/m was observed on the Vienna Ring Road. In addition, higher maximum values were measured for Zagreb Ring Road, Bologne Ring Road, Padoue Ring Road, Ljubljana Ring Road, and Beograd. The highest measured value was 14.59 V/m on the *Ez*-axis. The lowest *E_total_* value in all roads was 0.11 V/m. The lowest measured value was 0.047 V/m on the *Ez*-axis. Moreover, the data show that the exposure of car drivers and passengers during travel on European roads is below the ICNIRP reference level [17]. These data may be also helpful for the exposure assessment during in vitro and in vivo experiments.

With the comparison of existing measurement campaigns [7,8,9,10,12,13,14,15,18,19,20,21,39], we present results for the E-field in a complex environment due to the semi-resonant nature of the vehicle body. The presented information for the field strength of *Ex*, *Ey*, and *Ez* components serves as an indication for the orientation of the electric field, which is valuable for the design of both new absorbers of EMF for the car cabin and new flexible rectennas for EMF energy harvesting in vehicles.

Future studies will address the exposure assessment of car drivers and passengers during an ongoing voice call and data transmission in car generic wireless communications scenarios and new V2X communication scenarios as vehicle-to-vehicle (V2V), vehicle-to-infrastructure (V2I), vehicle-to-pedestrian (V2P), and vehicle-to-network (V2N) communications.

## Figures and Tables

**Table 1:** Settings of LSProbe 1.2 Field Probe Variant E.

Parameter	
Measurement mode	0
Frequency range (GHz)	0.03–8.2
Sampling rate (kS/s)	500
Sample timing	continuous
Sensitivity (mV/m)	60

**Table 2:** Driving distance (in km) on each road sector (between two points) in each country.

Country	Road Sector	Distance (km)	Total Distance (km)
Austria	P1 AT–P2 AT	107.0	219.0
	P2 AT–P3 AT	112.0	
Bulgaria	P1 BG–P2 BG	44.1	304.4
	P2 BG–P3 BG	84.3	
	P3 BG–P4 BG	179.0	
Croatia	P1 HR–P2 HR	67.3	303.3
	P2 HR–P3 HR	236.0	
Hungary	P1 HU–P2 HU	52.6	160.5
	P2 HU–P3 HU	58.7	
	P3 HU–P4 HU	49.2	
Italy	P1 IT–P2 IT	8.3	377.4
	P2 IT–P3 IT	100.0	
	P3 IT–P4 IT	69.7	
	P4 IT–P5 IT	53.4	
	P5 IT–P1 Sl	146.0	
Slovenia	P1 Sl–P2 Sl	58.6	186.5
	P2 Sl–P3 Sl	128.0	
Republic of Serbia	P1 RS–P2 RS	28.8	391.9
	P2 RS–P3 RS	125.0	
	P3 RS–P4 RS	36.9	
	P4 RS–P5 RS	69.8	
	P5 RS–P6 RS	50.3	
	P6 RS –P7 RS	32.4	
	P7 RS–P8 RS	48.7	

**Table 3:** Descriptive statistics of *E_total_* for all road sectors.

Country	Road Sector	N	Mean	Min	Median	Max
Austria	P1 AT–P2 AT	28,762	0.420	0.111	0.313	17.395
	P2 AT–P3 AT	14,684	0.326	0.111	0.308	1.353
Bulgaria	P1 BG–P2 BG	7905	0.358	0.111	0.250	7.144
	P2 BG–P3 BG	17,174	0.264	0.111	0.220	9.601
	P3 BG–P4 BG	34,404	0.261	0.111	0.226	11.332
Croatia	P1 HR–P2 HR	8421	0.330	0.111	0.237	4.940
	P2 HR–P3 HR	30,322	0.294	0.111	0.247	8.959
Hungary	P1 HU–P2 HU	11,067	0.250	0.111	0.224	5.329
	P2 HU–P3 HU	13,022	0.268	0.111	0.238	5.468
	P3 HU–P4 HU	9461	0.317	0.126	0.263	5.500
Italy	P1 IT–P2 IT	5358	0.329	0.111	0.275	3.803
	P2 IT–P3 IT	14,277	0.247	0.120	0.227	6.117
	P3 IT–P4 IT	6784	0.249	0.111	0.232	1.761
	P4 IT–P5 IT	18,241	0.289	0.111	0.247	7.209
	P5 IT–P1 Sl	18,550	0.318	0.111	0.255	9.941
Slovenia	P1 Sl–P2 Sl	12,187	0.323	0.111	0.275	5.606
	P2 Sl–P3 Sl	26,709	0.354	0.111	0.289	6.455
Republic of Serbia	P1 RS–P2 RS	4944	0.223	0.111	0.215	3.121
	P2 RS–P3 RS	10,073	0.270	0.120	0.231	3.519
	P3 RS–P4 RS	3452	0.251	0.126	0.234	3.935
	P4 RS–P5 RS	10,217	0.246	0.111	0.234	2.236
	P5 RS–P6 RS	7164	0.241	0.111	0.227	1.699
	P6 RS–P7 RS	5795	0.685	0.126	0.473	7.756
	P7 RS–P8 RS	6488	0.282	0.133	0.244	7.572

## Data Availability

The data presented in this study are available on request from the corresponding author.
